# Development of a home-based cognitive test for cognitive monitoring in subjective cognitive decline with high risk of Alzheimer’s disease

**DOI:** 10.1097/MD.0000000000033096

**Published:** 2023-03-03

**Authors:** Yun Jeong Hong, Si Baek Lee, Seong Hoon Kim, Myung Ah Lee, Jeong Wook Park, Dong Won Yang

**Affiliations:** a Department of Neurology, Uijeongbu St. Mary’s Hospital, The Catholic University of Korea, Uijeongbu, Korea; b Department of Neurology, Seoul St. Mary’s Hospital, The Catholic University of Korea, Seoul, Korea.

**Keywords:** Alzheimer’s disease, amyloid PET, amyloidosis, cognitive decline, neuroimaging biomarker, subjective cognitive decline

## Abstract

Subjective cognitive decline (SCD) indicates a self-perceived persistent cognitive worsening despite of normal performance in standard neuropsychological tests. Owing to its heterogeneity and potential risk of Alzheimer’s disease, baseline biomarkers to predict cognitive decline are important. In the present study, we developed a home-based cognitive test (HCT) to monitor cognitive changes regularly without visiting hospitals. This study aims to compare cognitive and biomarker trajectories during a 48-month period between amyloid positive SCD and amyloid negative SCD subjects.

**Methods:**

Data will be collected from a prospective observational cohort study conducted in South Korea. Eighty participants with SCD aged ≥ 60 years are eligible for the study. All participants undergo annual neuropsychological tests and neurological examinations, bi-annual brain MRI scans and plasma amyloid markers, and baseline florbetaben Positron Emission Tomography scans. The amyloid burden and regional volumes will be measured. Cognitive and biomarker changes will be compared between the amyloid-positive SCD and amyloid negative SCD groups. Validation would be performed to assess reliability and feasibility of HCT.

**Conclusions:**

This study would suggest a perspective on SCD in terms of cognitive and biomarker trajectories. Baseline characteristics and biomarker status might affect faster cognitive decline and future biomarker trajectories. In addition, HCT could be an alternative option of in-person neuropsychological tests to track cognitive changes without visiting hospitals.

## 1. Introduction

### 1.1. Background

Subjective cognitive decline (SCD) indicates a self-perceived persistent cognitive worsening despite of normal performance in standard neuropsychological tests.^[1]^ SCD is regarded as a risk group of dementia and Alzheimer’s disease (AD) and important as a target population for secondary preventions.^[2,3]^ However, due to its heterogeneity and uncertainty regarding future cognitive trajectories and underlying pathologies, SCD is not identical with preclinical stages of AD. Considering that subjects with SCD progress slowly, regular cognitive monitoring to assess clinical progressions in outpatient clinics are practically difficult. After worldwide pandemic of coronavirus 19, patients are more reluctant to vising hospitals. Therefore, baseline biomarker evaluations to predict faster cognitive declines and regular cognitive monitoring of the high-risk patients are clinically important.

We planned a cohort based longitudinal observational study to develop a home-based cognitive test (HCT) and assess cognitive and biomarker trajectories of SCD according to baseline biomarker status.

### 1.2. Study aims

The objective of this study is to assess whether amyloid-positive (A + ) SCD progress rapidly compared with amyloid negative SCD. Our second aim is to validate a newly developed HCT as an alternative cognitive monitoring tool without vising hospitals.

## 2. Methods

### 2.1. Study design

This study is a longitudinal observational cohort based study performed at a university-affiliated dementia clinic from May 2020 to December 2025. A total of 80 elderly participants with SCD who presented with a complaint of persistent cognitive decline are planned to be enrolled at baseline in a single center and undergo annual follow up evaluations during 48 months (see Table 1).

**Table 1:** Data collection.

Assessments	Screening	Baseline	12/36 months	24 months	48 months
Eligibility	**√**				
Demographics	**√**				
Comorbidity	**√**	**√**			
Concomitant medication	**√**	**√**	**√**	**√**	**√**
Blood chesmistry	**√**				
Plasma amyloid beta		**√**		**√**	
SNSB	**√**		**√**	**√**	**√**
Self-report questionnaire		**√**	**√**	**√**	**√**
Brain MRI	**√**			**√**	
Florbetaben PET		**√**			
Physical examination	**√**	**√**	**√**	**√**	**√**
Vital signs		**√**	**√**	**√**	**√**
Neurologic examination	**√**	**√**	**√**	**√**	**√**
HCT*		**√**	**√**	**√**	**√**
Physician’s judgements of progression			**√**	**√**	**√**

HCT = home-based cognitive test, PET = positron emission tomography, SNSB = Seoul neuropsychological screening battery.

* Home-based cognitive tests (HCT) are performed every 6 months during the study period.

First, we have developed a HCT (score range 0–30, higher scores indicate better cognitive function) composed of 20 questions that can be assessed using phone-calls. Second, neuropsychological tests, blood samplings, physical and neurologic examinations, brain MRIs, florbetaben positron emission tomography (PET) scans, self-report questionnaires are evaluated at baseline. Third, all participants undergo annual follow up neuropsychological tests, physical and neurologic examinations, physician’s history taking to judge clinical progression. All participants should undergo bi-annual brain MRI scans and plasma amyloid beta markers until the end of the study (Table 1). The first participant was enrolled in June 2020, and remaining participants are being recruited.

### 2.2. Study registration

This study trial has been registered on the Clinical Research Information Service (http://cris.nih.go.kr) with an ID of No. KCT0005254.

### 2.3. Participants

Individuals who were diagnosed as SCD were eligible for the study. Subjects who visited the hospital due to persistent cognitive worsening and were diagnosed with SCD after dementia work-ups are consecutively recruited. The dementia work up included detailed neuropsychological test battery, MRI, and routine blood sampling for syphilis, thyroid function, vitamin B deficiencies, and apolipoprotein epsilon genotyping. The inclusion criteria were as follows (Table 2): Age ≥ 60 years of age; Existence of persistent self-reported cognitive complaints; Normal performance (above −1.0 standard deviation [SD] of norms) on all subtests of neuropsychological test battery named Seoul Neuropsychological Screening Battery (SNSB);^[4]^ Clinical dementia rating (CDR) score of 0;^[5]^ Agreement to participate in the study and could visit the hospital for annual evaluations. The exclusion criteria were the following: Mild cognitive impairment (MCI) or dementia; Brain lesions known to cause cognitive impairment (tumor, stroke, or subdural hematoma); Any neurological disorders such as Parkinson’s disease, Huntington’s disease, epilepsy, or normal pressure hydrocephalus; Major psychiatric disorders such as uncontrolled depression, schizophrenia, alcoholism, or drug dependency; Abnormal blood laboratory findings such as abnormal thyroid function, low vitamin B12 or low folate, or positive syphilis serology, and; Hearing loss that is impossible to perform phone-based cognitive tests.

**Table 2:** Eligibility criteria.

Inclusion criteria
age ≥ 60 yr of age
existence of persistent self-reported cognitive complaints
normal performance (≥−1.0 standard deviation of norms) on all subtests of SNSB
CDR score of 0
agreement to participate in the study and could visit the hospital for annual evaluations
**Exclusion criteria**
MCI or dementia
brain lesions known to cause cognitive impairment (tumor, stroke, or subdural hematoma)
any neurological disorders such as Parkinson’s disease, Huntington’s disease, epilepsy, or normal pressure hydrocephalus
major psychiatric disorders such as uncontrolled depression, schizophrenia, alcoholism, or drug dependency
abnormal blood laboratory findings such as abnormal thyroid function, low vitamin B12 or low folate, or positive syphilis serology
hearing loss that is impossible to perform phone-based cognitive tests

CDR = clinical dementia rating, MCI = mild cognitive impairment, SNSB = Seoul neuropsychological screening battery.

### 2.4. Recruitment

**Figure 1.**
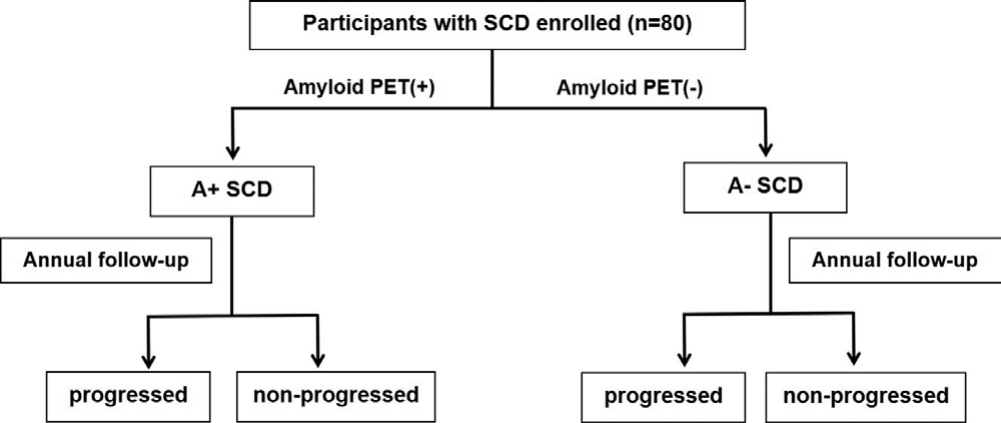
Study flowchart.

We will recruit 80 participants with SCD (Fig. 1). The researcher explains the aim and the methods of the study and will obtain informed consent from potential participants before the collection of medical information.

### 2.5. Intervention

No study related pharmacological/ non pharmacological interventions are planned. For participants who progressed to dementia, regardless of amyloid positivity, they can be treated with acetylcholinesterase inhibitors according to physician’s practical decision.

### 2.6. Neuropsychological tests

SNSB includes Korean version of the mini-mental state examination (K-MMSE),^[6]^ CDR, Korean version of activities of daily living,^[7]^ attention (digit span test), language (Boston naming test, tests for comprehension/repetition/fluency), visuospatial function (Rey Complex Figure Test), verbal and visual memory function (Seoul Verbal Learning Test and Rey complex figure test recall test), and frontal executive function (contrasting program, go-no-go, Controlled Oral Word Association Test, and Stroop test).^[4]^ Age, sex, and education-specific norms based on normal controls were used to interpret the SNSB results. Scores ≥ 16th percentile, which were compared to–1 SD of the norm, were defined as normal. Severity of the cognitive complaints were assessed using a self-rated scale named cognitive failures questionnaire (total score 0–100, higher total score means more cognitive complaints).^[8]^ Using a self-report questionnaire, “informant also report a cognitive decline of the participant,” “subjective concern about the cognitive decline,” and “symptom’s onset after 65 years of age” were assessed at baseline and follow-up evaluations.

### 2.7. Outcome measures

The primary outcome measure is K-MMSE score changes at the endpoint. The secondary outcome measures include demographic data, baseline and follow-up SNSB subdomain scores, self-report questionnaires, HCT scores, brain MRI markers, florbetaben PET positivity, quantitative regional amyloid depositions using PET scans, and clinical progression rates.

All participants will undergo baseline neurologic examinations, neuropsychological tests named SNSB, brain MRIs, blood labs, and florbetaben PET scans for amyloid depositions. PET findings are interpreted using a visual rating scale named brain amyloid plaque load and rated as positive amyloidosis with a brain amyloid plaque load score of 2/3.^[9]^ Quantitative neuroimaging analysis will be performed.

At baseline, questionnaires for SCD, amyloid PET scans, brain MRIs including 3 dimensional-T1 imaging, plasma amyloid beta values are examined. Telephone-based HCT at home are performed every 6 months during the study period. Annual follow-up evaluations include detailed neuropsychological tests, physical and neurologic examinations, and physician’s assessments for clinical progression. Brain MRI and plasma amyloid beta values are assessed at baseline and 24 months later (Table 1).

Clinical progression to mild cognitive impairment or dementia will be assessed at the final visit. The cognitive tests were administered by a trained neuropsychologist. Participants with CDR score ≥ 0.5 or Korean version of activities of daily living score ≥ 0.43 were considered to have progressed to MCI or dementia.

### 2.8. Neuroimaging analysis

Brain MRI are performed using a 3.0-Tesla scanner (GE Medical Systems, Milwaukee, WI), including fluid attenuated inversion recovery (FLAIR), susceptibility weighted image, and 3-dimensional (3D) T1-weighted images. The white matter hyperintensities (WMHs) are rated using a visual rating scale of axial FLAIR images. In brief, periventricular WMHs and deep WMHs are evaluated separately and rated as minimal (grade 1), moderate (grade 2), or severe (grade 3).^[10]^ Lacunes are defined as small lesions (3–15 mm in diameter), hyperintense on *T*2-, and hypointense on *T*1-weighted images, with a perilesional halo on FLAIR.^[11]^ Cerebral cortical microbleeds are defined as round and homogeneously low-signal lesions <10 mm in diameter on susceptibility weighted image.^[11]^ Hippocampal atrophy is rated on coronal *T*1-weighted images using Scheltens visual rating scale.^[12]^ The number of lacunes, number of microbleeds, degree of WMH, and degree of hippocampal atrophy will be measured by a trained neurologist blinded to the data.

Florbetaben (18F) PET scans are acquired following the standardized protocol.^[13]^ Using PET scans, a whole brain visual interpretation is performed by a trained doctor in nuclear medicine who is blinded t the patient diagnosis. In addition, quantitative neuroimaging analyses are performed using PET scans and MRI 3D-*T*1 images. First, amyloid depositions were assessed using MATLAB version 2013a and SPM8 (http://www.fil.ion.ucl.ac.uk/spm/software/spm8). Individual 3D *T*1-weighted MRI scans are estimated and co-registered into corresponding PET images. A volume-based template, incorporating 90 regions-of-interest, is aligned to individual *T*1-weighted MRI scans. The voxels of florbetaben PET images were scaled using the mean uptake value in the cerebellar gray matter to calculate the standardized uptake value ratio (SUVR), and partial volume corrections are performed. The mean SUVR values are calculated as a global SUVR. Second, the MRI volumetric analysis are performed using AQUA 2.0 program (Neurophet, South Korea). The details of the MRI segmentation and data analysis were described elsewhere.^[14]^ A normative dataset is obtained using the East-Asian dataset described in a previous study,^[15]^ and the adjusted volume (z score) corrected with total intracranial volume, age, and sex is measured.

### 2.9. Plasma amyloid beta values

Plasma amyloid beta values are measured using the Multimer Detection System-oligomeric Aß (MDS-OAß) method.^[16]^ In brief, the inBloodTM™ OAß test (People Bio Inc., Gyeonggi-do, Republic of Korea) will be used to quantify MDS-OAß values in heparin vacutainer tubes. Higher values indicate more amyloid oligomeric tendencies with vigorous amyloidosis. Plasma amyloid beta values are assessed at baseline and 24 months.

### 2.10. Sample size calculation

We assumed that a 3.5-point difference in K-MMSE assessment scores (the primary outcome measure between the A + SCD and SCD groups) after 48 months would be significant. With an alpha value set at 0.05, a beta value of 0.9 (90% power), and a SD value of 4.5 for change, a total of 36 participants were required for each study group. Assuming a discontinuation rate of 10% during the 48-month study, the total calculated sample size required for the study was 80 participants.

### 2.11. Statistical analysis

Independent *t* test or nonparametric Mann–Whitney *U* test (based on normal distribution patterns) is used for comparison of continuous variables such as baseline demographics and clinical characteristics between A + SCD and A − SCD participants. Chi-square tests will be used to compare categorical variables between the 2 groups. analysis of covariance corrected for baseline scores would be used to compare cognitive changes between the 2 groups. All statistical analyses are performed using SPSS (version 18.0; SPSS Inc, Chicago, IL). *P* values < 0.05 is considered to indicate statistically significant differences.

### 2.12. Data collection and study ethics

Data will be processed de-identified and will be recorded on electronic case report forms (eCRF). Access to the eCRF links will be restricted to the study investigators. If a participant withdraws from the study, reasons for the withdrawal will be recorded and all attempts will be made to collect endpoint data. Reasons. Access to the participant’s personal information will be restricted to the study coordinator and investigators involved in screening. This investigator-initiated clinical trial received funding support from National Research Foundation of Korea, but this sponsor is neither involved in the study design nor its proposed operations. The research proposal was approved by the Institutional Review Boards at each of the participating institutions (IRB file No. UC20ONSI0052 at Uijeongbu St. Mary’s Hospital), written informed consent will be obtained for all study participants by the principal investigator.

### 2.13. Dissemination

Our study results will be disseminated through presentations and publications. Publication of the results will be publicated as a journal article. All manuscripts will be authored by the study team and authorship will follow the established publication guidelines such as those of the International Committee of Medical Journal Editors.

## 3. Discussion

In this study, we aim to conduct a longitudinal observational study to assess cognitive and biomarker changes in Korean participants with SCD over the course of 48 months. We will assess whether SCD participants showed different cognitive and biomarker trajectories according to biomarker status. We hypothesize that A + SCD participants would show more cognitive decline and atrophic changes after 48 months. Baseline neurodegenerative changes and amyloid burden are expected to be related with cognitive trajectories in SCD. In particular, neurodegenerations combined with sufficient amyloid depositions above the threshold level would show rapid cognitive progression in SCD participants because existence of the 2 key pathologies indicate later stages of preclinical AD. In addition, in an effort to monitor cognitive declines in SCD participants, we have developed a new HCT as an alternative option of hospital-based in-person neuropsychological tests. Considering that subjects with normal cognition are still reluctant to visiting hospitals for regular in-person cognitive tests and this population generally show lower annual progression rates (5–10% annually) compared with those with MCI/ dementia,^[2,3,17]^ regular cognitive monitoring at home would be clinically helpful to detect cognitive changes in SCD subjects without visiting hospitals for the cognitive tests.

We are now conducting recruitments of the participants and baseline database will be completed by 2023. Despite of a few limitations that other biomarkers assessing combined tau, alpha synuclein, or TAR DNA-binding protein 43 pathologies are not evaluated, our study have several strengths as followings: Participants are consecutively recruited in South Korea using a comprehensive neuropsychological test battery and undergo various biomarker evaluations, allowing us to clarify cognitive and biomarker trajectories of SCD participants. All participants undergo baseline amyloid PET scans, regular brain MRI scans, and plasma amyloid beta markers to quantitatively measure amyloid burden and neurodegenerative changes.

## Author contributions

**Conceptualization:** Yun Jeong Hong.

**Data curation:** Si Baek Lee, Seong Hoon Kim, Myung Ah Lee, Jeong Wook Park, Dong Won Yang.

**Formal analysis:** Yun Jeong Hong.

**Funding acquisition:** Yun Jeong Hong.

**Investigation:** Yun Jeong Hong, Si Baek Lee, Seong Hoon Kim, Myung Ah Lee, Jeong Wook Park, Dong Won Yang.

**Methodology:** Si Baek Lee, Seong Hoon Kim, Myung Ah Lee, Jeong Wook Park, Dong Won Yang.

**Project administration:** Yun Jeong Hong.

**Supervision:** Jeong Wook Park, Dong Won Yang.

**Writing – original draft:** Yun Jeong Hong.

**Writing – review & editing:** Dong Won Yang.
